# Production and characterization of films from nanocomposite coating of sunflower seeds

**DOI:** 10.1186/1753-6561-8-S4-P225

**Published:** 2014-10-01

**Authors:** Andriele Mendonça Barbosa, Klebson Silva Santos, Malone Santos Pinheiro, Isabelle Souza de Mélo Silva, Flávia Manuella Ribeiro de Mendonça, Fernando Mendonça Diz, Luzia Nilda Tabosa Andrade, Resende Yane Tainara Menezes, Rafaela Andrade Fonseca, Juliana Cordeiro Cardoso, Maria Lucila Hernandez Macedo, Francine Ferreira Padilha

**Affiliations:** 1 Programa de Pós, Graduação em Biotecnologia Industrial, Universidade Tiradentes, Avenida Murilo Dantas, 300, Farolândia, CEP 49.032-490, Aracaju, SE, Brazil; 2 Programa de Doutorado da Rede Nordeste de Biotecnologia, Universidade Tiradentes, Avenida Murilo Dantas, 300, Farolândia, CEP 49.032-490, Aracaju, SE, Brazil; 3 Laboratório de Biomateriais, Instituto de Tecnologia e Pesquisa, Avenida Murilo Dantas, 300, Farolândia, CEP 49.032-490, Aracaju, SE, Brazil

## Background

The culture of sunflower plants presents a great economic relevance. Sunflower's seed stand out for your high high concentration of oil, which is used in human and animal feeding, and currently, as source of raw material to obtain biodiesel. However, about 12% of worldwide sunflower production is lost per year due to diseases occurrence, being this, the most limiting factor to the culture in most producing regions. The main diseases is Alternaria stain, which affects sunflower culture, are transmitted through the seeds. The coating technique has been researched by food and agricultural industry, once the coating of seeds with biodegradable films enable an uniform application of chemical and biological agents against the attack of pathogens and phytopathogens. This action reduces the impact over the environment and farmer's health. Additives incorporated in biodegradable films production used in the coaters have as function to protect the seeds against the attack of microorganisms. The aim of this work was to develop a nanobiocomposite of metal-polymer to coat the sunflower seeds.

## Methods

Tests were performed according to the experimental delineation: the chosen polymer (pectin, hydrolyzed collagen and xanthan gum); plasticizers (propylene glycol and glycerol) and the addictive (propolis and metal-polysaccharide nanocomposite). The films were prepared by casting technique. This method consists of a method used for food products, that require a uniform coating on an uneven surface. After the immersion, the excess coating material is drained from the product and then it is dried or left to stand to solidify. The films were evaluated against their mechanics properties and activity against Alternaria sp.. The coating in the sunflower seeds was observed by scanning electron microscopy. The propylene glycol-plasticized films were developed experimentally, and the best-performing formulation (2% pectin and 0.6% propylene glycol) was selected for HPE incorporation. The film with 5% HPE had antifungal activity against the phytopathogen and was used to coat the sunflower seeds.

## Results and conclusion

The incorporation of HPE improved the mechanical properties of the film and did not change other film characteristics. Photomicrographs were obtained of the films and coated seeds, this procedure showed their morphological characteristics and total seed coating when the film is incorporated. The films containing metal-polymer presented a similar behave as the pectin coated films, having a superior biological activity comparing with films containing red propolis, showing, therefore, an excellent biotechnological potential to seeds coating technology.

